# Incidence and Determinants of Symptomatic and Asymptomatic SARS-CoV-2 Breakthrough Infections After Booster Dose in a Large European Multicentric Cohort of Health Workers-ORCHESTRA Project

**DOI:** 10.1007/s44197-023-00139-8

**Published:** 2023-07-22

**Authors:** Stefano Porru, Maria Grazia Lourdes Monaco, Gianluca Spiteri, Angela Carta, Gulser Caliskan, Concepción Violán, Pere Torán-Monserrat, Luigi Vimercati, Silvio Tafuri, Paolo Boffetta, Francesco Saverio Violante, Emma Sala, Emanuele Sansone, Fabriziomaria Gobba, Loretta Casolari, Andreas Wieser, Christian Janke, Adonina Tardon, Marta Maria Rodriguez-Suarez, Filippo Liviero, Maria Luisa Scapellato, Marco dell’Omo, Nicola Murgia, Dana Mates, Violeta Claudia Calota, Jozef Strhársky, Mariana Mrázová, Enrico Pira, Alessandro Godono, Greta Camilla Magnano, Corrado Negro, Giuseppe Verlato, Orchestra WP5 Working Group, Maria Diletta Pezzani, Evelina Tacconelli, Davide Gibellini, Virginia Lotti, Lucí Amalia Carrasco-Ribelles, Eva María Martínez Cácers, Julia Garcia Prado, Noemí Lamonja-Vicente, Luigi De Maria, Pasquale Stefanizzi, Stefania Sponselli, Antonio Caputi, Mahsa Abedini, Giorgia Ditano, Shuffield S. Asafo, Giulia Collatuzzo, Giuseppe De Palma, Alberto Modenese, Giorgia Rossi, Francesca Glieca, Daniela Vivoli, Michael Hoelscher, Noemi Castelletti, Christina Reinkemeyer, Thu Giang Le Thi, Guillermo Fernandez-Tardon, Pedro Ignacio Arcos-Gonzalez, Francisco José Jimeno-Demuth, Carmen Natal-Ramos, Angelo Moretto, Paola Mason, Sofia Pavanello, Anna Volpin, Giacomo Muzi, Angela Gambelunghe, Ilenia Folletti, Tiziana Fiordi, Andra Neamtu, Ovidiu Perseca, Catalin Alexandru Staicu, Angelica Voinoiu, Eleonóra Fabiánová, Roman Nedela, Jana Oravec Bérešová, Lenka Palcová, Ihab Mansour, Catalina Ciocan, Andrea Franceschi, Francesca Larese Filon, Luca Cegolon

**Affiliations:** 1 Section of Occupational Medicine, Department of Diagnostics and Public Health, University of Verona, 37134 Verona, Italy; 2 Occupational Medicine Unit, University Hospital of Verona, 37134 Verona, Italy; 3 Unit of Epidemiology and Medical Statistics, Department of Diagnostics and Public Health, University of Verona, 37134 Verona, Italy; 4 Institut Universitari d’Investigació en Atenció Primària Jordi Gol (IDIAP Jordi Gol), Unitat de Suport a la Recerca Metropolitana Nord, Mare de Déu de Guadalupe 2, Planta 1ª, Mataro, 08303 Barcelona, Spain; 5 Germans Trias i Pujol Research Institute (IGTP), Camí de les Escoles, S/N, Badalona, 08916 Barcelona, Spain; 6 Interdisciplinary Department of Medicine, University of Bari, 70124 Bari, Italy; 7 Department of Medical and Surgical Sciences, University of Bologna, Bologna, Italy; 8 Unit of Occupational Health, Hygiene, Toxicology and Prevention, University Hospital ASST Spedali Civili, 25123 Brescia, Italy; 9 Department of Medical and Surgical Specialties, Radiological Sciences and Public Health, Unit of Occupational Health and Industrial Hygiene, University of Brescia, 25123 Brescia, Italy; 10 Department of Biomedical, Metabolic and Neural Sciences, University of Modena and Reggio Emilia, 41125 Modena, Italy; 11 Health Surveillance Service, University Hospital of Modena, 41125 Modena, Italy; 12 Division of Infectious Diseases and Tropical Medicine, University Hospital, LMU Munich, 80802 Munich, Germany; 13 German Center for Infection Research (DZIF), Partner Site Wieser@mvp.Munich, 81377 Munich, Germany; 14 Fraunhofer Institute for Translational Medicine and Pharmacology ITMP, Immunology, Infection and Pandemic Research, 80799 Munich, Germany; 15 Max Von Pettenkofer Institute, Faculty of Medicine, LMU Munich, 80336 Munich, Germany; 16 University of Oviedo, Health Research Institute of Asturias (ISPA) and CIBERESP, Asturias, Spain; 17 HUCA (SESPA), University of Oviedo, and Health Research Institute of Asturias (ISPA), Asturias, Spain; 18 Department of Cardiac Thoracic Vascular Sciences and Public Health, University of Padova, Padua, Italy; 19 University Hospital of Padova, 35128 Padua, Italy; 20 Section of Occupational Medicine, Respiratory Diseases and Toxicology, Department of Medicine and Surgery, University of Perugia, 06123 Perugia, Italy; 21 Department of Environmental and Prevention Sciences, University of Ferrara, 44121 Ferrara, Italy; 22 National Institute of Public Health, Bucharest, Romania; 23 Medical Microbiology Department, Regional Authority of Public Health, 97556 Banská Bystrica, Slovakia; 24 Public Health Institute, St. Elizabeth University of Health and Social Work, 81106 Bratislava, Slovakia; 25 Department of Public Health and Pediatrics, University of Torino, 10126 Turin, Italy; 26 Department of Medical Sciences, Unit of Occupational Medicine, University of Trieste, 34129 Trieste, Italy

**Keywords:** SARS-CoV-2, COVID-19 breakthrough infections, COVID-19 vaccine, Booster vaccination, Booster dose, Vaccine effectiveness

## Abstract

**Background:**

SARS-CoV-2 breakthrough infections (BI) after vaccine booster dose are a relevant public health issue.

**Methods:**

Multicentric longitudinal cohort study within the ORCHESTRA project, involving 63,516 health workers (HW) from 14 European settings. The study investigated the cumulative incidence of SARS-CoV-2 BI after booster dose and its correlation with age, sex, job title, previous infection, and time since third dose.

**Results:**

13,093 (20.6%) BI were observed. The cumulative incidence of BI was higher in women and in HW aged < 50 years, but nearly halved after 60 years. Nurses experienced the highest BI incidence, and administrative staff experienced the lowest. The BI incidence was higher in immunosuppressed HW (28.6%) vs others (24.9%). When controlling for gender, age, job title and infection before booster, heterologous vaccination reduced BI incidence with respect to the BNT162b2 mRNA vaccine [Odds Ratio (OR) 0.69, 95% CI 0.63–0.76]. Previous infection protected against asymptomatic infection [Relative Risk Ratio (RRR) of recent infection vs no infection 0.53, 95% CI 0.23–1.20] and even more against symptomatic infections [RRR 0.11, 95% CI 0.05–0.25]. Symptomatic infections increased from 70.5% in HW receiving the booster dose since < 64 days to 86.2% when time elapsed was > 130 days.

**Conclusions:**

The risk of BI after booster is significantly reduced by previous infection, heterologous vaccination, and older ages. Immunosuppression is relevant for increased BI incidence. Time elapsed from booster affects BI severity, confirming the public health usefulness of booster. Further research should focus on BI trend after 4th dose and its relationship with time variables across the epidemics.

**Supplementary Information:**

The online version contains supplementary material available at 10.1007/s44197-023-00139-8.

## Introduction

The vaccination campaign against SARS-CoV-2 started at the end of 2020 and showed high effectiveness in preventing infection and severity of disease in subjects who had received a complete vaccination course [1–4]. However, the protection against infection may wane, in particular after the surge of the variant of concerns [5]; therefore, as of autumn 2021, the World Health Organization recommended a booster dose to the general population, giving priority to older and immunocompromised people, as well as health workers (HW) [6].

The Omicron variant first appeared in November 2021 in South Africa and quickly spread around the world. Chenchula et al. analysed 27 studies, which evaluated the effectiveness of booster dose compared to none or primary course only [5]. The results confirmed that the protection of the primary course against infection waned to 33% during the Omicron wave, as well as the risk of reinfection in subjects previously infected with the original viral strain increased compared to those who had been infected with the Delta and Beta variants. On the other hand, subjects vaccinated with booster doses were 81% less likely to be hospitalized than those unvaccinated. Furthermore, the neutralizing efficiency against Omicron increased by 1.4–100 times after administering the third dose, although it remained much lower than that against the Delta variant. The booster dose administration also revealed improved effectiveness in preventing symptomatic cases from Omicron and Delta variants [5]. These results were confirmed in the meta-analysis by Meggiolaro et al. [7].

Indeed, compared to unvaccinated, the risk of Omicron symptomatic infection was 24% and 69% lower in subjects who had undergone primary vaccination or also a booster dose, respectively. The risk reduction was larger for hospitalization, but the difference between people receiving primary vaccination alone and the booster dose persisted (50% and 88%, respectively). Moreover, the protective effect of the primary course against symptomatic infection decreased faster than against hospitalization. On the other hand, no decay was reported for effectiveness five months after the booster dose administration [7]. In a short-term follow-up study (median 39 days), carried out on 1,928 HW, tested for SARS-CoV-2 with RT-PCR every 14 days, an Adjusted Hazard Ratio (AHR) of 0.07 was reported comparing booster immunized vs no booster immunized. No relevant differences were found in symptomatic and asymptomatic infections (AHR = 0.07 and 0.008, respectively) [8]. In contrast, a large population study with longer follow-up reported that the booster dose effectiveness decreases over time. Indeed, the relative vaccine effectiveness for symptomatic infection diminished from 41.4% 7–30 days after the administration to 12.2% over 120 days later. The decline was more complete for mild disease (from 7.9% to negative values) than moderate (from 56.0 to 27.1%) or severe (from 80.9 to 63.4%) diseases [9]. A previous study in the ORCHESTRA Project investigated SARS-CoV-2 infection risk factors among primary-course vaccinated HW [4]. Results showed that the risk of infection varied before and after the vaccination. Indeed, nurses and other workers involved in patients’ care were at higher risk before the vaccination, while no significant differences were highlighted after vaccination.

While there was no association between age and incidence before vaccination, vaccine administration appeared more protective as age increased. Lastly, previous infected HW had an Odds Ratio (OR) of 0.43 compared to SARS-CoV-2 naïve HW [10]. Vivaldi et al., in a large prospective population-based study, identified risk factors for SARS-CoV-2 infection after primary and booster vaccinations. They found a lower risk of breakthrough infections (BI) in fully vaccinated workers, previously infected, aged over 65, and employed in frontline health or social care. Previous infection and older age were confirmed as protective factors in boosted participants, while heterogeneous vaccination versus BNT16b2b2 administration increased the risk both in the primary course and booster dose settings [11].

The aim of this study is to analyse the incidence of SARS-CoV-2 BI, both symptomatic and asymptomatic, after booster dose and their demographic, occupational and clinical determinants, among a large multicentric European cohort involving over 60,000 HW.

## Materials and Methods

### Design and Setting

A multicentre retrospective cohort study of HW from 14 European centres involved in the ORCHESTRA project was performed [12]. Data were collected from various healthcare settings (University Hospital, Primary healthcare centres, and nursing homes), in Germany (Munich), Italy (Bari, Bologna, Brescia, Modena, Padua, Perugia, Torino, Trieste, and Verona), Romania, Slovakia, and Spain (Northern Barcelona and Oviedo). This study was reported according to the STROBE (Strengthening the Reporting of Observational Studies in Epidemiology) guidelines [13]. The follow-up started 14 days after the booster dose, administered from September 2021 to the 17th of May 2022, and ended on the 31st of May 2022 or at the time of BI. The median follow-up was 176 days (p25–p75 = 165–195 days).

### Vaccination, Case Definition, and Inclusion Criteria

HW enrolled in a continuous follow-up since the pandemic's beginning was included.

Booster vaccination was defined as a complete primary vaccination series followed by a further dose at least six months later [14]. The inferential analysis was conducted on HW that had received, from September 2021 to June 2022, the booster dose of BNT162b2 (Pfizer-BioNTech), mRNA-1273 (Moderna), ChAdOx1 nCoV-19 (Oxford–AstraZeneca), and Ad26.COV2.S (Johnson). Both homologous boosters (same as the primary vaccine) and heterologous boosters (different from the primary vaccine) in fully vaccinated recipients were considered. In this study, the homologous booster included only BNT162b2 vaccine doses.

BI post-booster dose was defined as PCR-detected SARS-CoV-2 infection, occurring after the vaccine booster dose. BI after the booster dose included all COVID-19 infections at least 14 days after the booster dose, following the definition of BI after full vaccination previously used [4].

### Outcome and Data Collection

The incidence of SARS-CoV-2 BI post-booster dose and the main characteristics of infected subjects (sex, age, job title, previous infections, comorbidities, and body mass index—BMI) were investigated. Clinical and laboratory data were collected using a standardized data collection form.

SARS-CoV-2 infection was diagnosed by positive real-time reverse-transcriptase polymerase chain reaction (RT-PCR), performed using various commercially available assays in different clinical laboratories. Each centre adopted a different and variable timing for screening programmes through nasopharyngeal swabs except for Padua Hospital (which adopted RT-PCR on saliva samples since the beginning of 2022) [15] and Munich healthcare settings (which assessed self-reported RT-PCR-confirmed infections by questionnaire and web-app), according to epidemiological contexts and local regulations.

### Statistical Analysis

The significance of the association between 3-dose vaccination and potential determinants was investigated by chi-square test or Fisher’s exact test for categorical variables and by *t* test for continuous variables. The following determinants were considered: gender (men/women), age class (< 30, 30–39, 40–49, 50–59, ≥ 60 years), job title (physician, nurse, other HW, technician, administrative), comorbidities and in particular immunodepression (yes/no), centres and BMI (in kg/m^2^).

Cumulative incidence of BI was computed with the corresponding 95% confidence interval (CI), calculated by the Clopper–Pearson method. Significance of the association between BI and potential determinants was evaluated by a chi-square test. The main determinants considered were gender, age classes, job title, previous infection (No, yes before 1st dose, yes after 1st dose and before booster dose), type of vaccines (homologous, heterologous, other), and comorbidities (yes/no). Of note, information on comorbidities was available on 26.8% of HW (*n* = 17,020).

Multivariable analysis was accomplished by a two-level logistic regression model, where SARS-CoV-2 infection after the booster dose, coded as yes/no, was the response variable, and sex, age classes, job title, previous infection, and type of vaccine administered were the potential determinants. In subsequent analysis, BI were coded in three levels (none, asymptomatic and symptomatic), and the same determinants, also including immunodepression, were investigated by a two‐level multinomial logistic regression model [16].

In addition, it was verified whether the presence of symptoms during BI was affected by the time elapsed between the booster dose of vaccine and BI onset. A chi-square for trend was used to evaluate the significance of the association between symptoms (yes/no) and time lag, coded in quartiles (14–63, 64–93, 94–130, and 131–238 days). In addition, multivariable analysis was accomplished by a two-level logistic regression model. Symptoms (yes/no) during BI were the response variable, time lag was the explanatory variable, while sex, age classes, job title, previous infection, and type of vaccine were included as potential confounders. Of course, the latter analysis was restricted to HW with BI.

Results were synthesized through the OR for logistic models and through the relative risk ratios (RRR) for the multinomial model. In two-level models, level‐1 units were HW, who in turn were nested into participating centres (level-2 units). The level of statistical significance was set at 5%, and CI were calculated at 95%. Statistical analyses were performed using STATA software, release 17.0 (StataCorp, College Station, TX, USA).

## Results

### Population Under Study

The present study considered 80,463 HW, enrolled in 14 healthcare settings (Table 1S). Analysis of determinants was carried out on 63,516 (78.9%) HW vaccinated with booster dose. The proportion of HW administered the booster dose was higher in men than women, in people younger than 60 years, in nurses, and in people with comorbidities. The proportion of booster dose administration peaked in immunosuppressed HW (92%). A large variability in vaccination was observed across centres, as the proportion of booster dose administration ranged from 31.1% in Oviedo (Spain) to 99.3% in Munich (Germany) (Table 1).

**Table 1:** Sample characteristics comparing booster-dose vaccinated health workers versus those who had not received a booster dose

	Booster dose vaccinated (*n* = 63,516)	Not receiving booster dose (*n* = 16,947)	*P* value
Gender			0.002
Men	19,344 (79.6%)	4,950 (20.4%)	
Women	44,124 (78.6%)	11,984 (21.4%)	
Age			< 0.001
< 30	8,188 (81.3%)	1,882 (18.7%)	
30–39	14,187 (79.0%)	3,781 (21.0%)	
40–49	13,239 (79.6%)	3,399 (20.4%)	
50–59	18,944 (81.5%)	4,305 (18.5%)	
≥ 60	8,958 (71.5%)	3,580 (28.6%)	
Job title			< 0.001
Physicians	17,209 (83.6%)	3,382 (16.4%)	
Nurse	19,444 (86.1%)	3,131 (13.9%)	
Other Health Workers	9,333 (82.6%)	1,968 (17.4%)	
Technicians	5,530 (85.0%)	976 (15.0%)	
Administrative	4,462 (83.6%)	874 (16.4%)	
Comorbidity			< 0.001
No	14,191 (65.9%)	7,353 (34.1%)	
Yes	2,829 (79.6%)	726 (20.4%)	
Immunodepression			< 0.001
No	13,526 (85.3%)	2340 (14.7%)	
Yes	688 (92.0%)	60 (8.0%)	
Centre			< 0.001
Turin	9,816 (91.3%)	932 (8.7%)	
Brescia	8,212 (92.2%)	691 (7.8%)	
Verona	5,535 (86.8%)	842 (13.2%)	
Padua	6,548 (76.9%)	1,963 (23.1%)	
Trieste	6,096 (76.6%)	1,863 (23.4%)	
Modena	5,003 (95.0%)	264 (5.0%)	
Bologna	7,054 (92.9%)	543 (7.2%)	
Perugia	2,191 (57.6%)	1,614 (42.4%)	
Bari	5,641 (91.0%)	555 (9.0%)	
Oviedo	2,559 (31.1%)	5,667 (68.9%)	
Northern Barcelona	513 (60.5%)	335 (39.5%)	
Munich	3,259 (99.3%)	23 (0.7%)	
Slovakia	500 (46.6%)	572 (53.4%)	
Romania	589 (35.2%)	1,083 (64.8%)	
BMI (kg/m^2^), median, p25–p75	23.4 (21.1–26.4)	24.5 (21.9–27.8)	< 0.001

*P* values were computed by the Fisher’s exact test or chi-square test for categorical variables and Wilcoxon–Mann–Whitney rank-sum test for BMI

As regards type of vaccine, most HW (*n* = 54,710, 87.5%) received BNT162b2 (Pfizer–BioNTech) and 10.9% (*n* = 6826) different types of vaccine (heterologous vaccine). A negligible proportion received other types of vaccine: 1.5% (*n* = 943) mRNA-1273 (Moderna), 0.03% (*n* = 16) ChAdOx1 nCoV-19 (Oxford–AstraZeneca), 0.005% (*n* = 3) Ad26.COV2.S (Johnson) (Table 2S).

The study population of 63,516 HW was mainly represented by the Italian cohorts (88.3%). Most HW were female 44,124 (69.5%). Nurses (*n* = 19,444; 34.7%) and physicians (*n* = 17,209; 30.7%) were the most prevalent job titles.

### Determinants of Breakthrough Infection After the Booster Dose

In the 14 centres, 13,093 HW had BI after booster dose vaccine, yielding a cumulative incidence of 20.6% (95% CI 20.3–20.9%). Cumulative incidence was the highest in Slovakia 29.0% (95% CI 25.1–33.2%) and in Northern Italy: 31.1% (95% CI 29.9–32.3%) in Verona, 29.7% (28.6–30.8%) in Padua, and 28.9% (27.8–30.0%) in Trieste (Table 1S).

The association between main demographic and clinical characteristics and BI after the booster dose is presented in Table 2.

**Table 2:** Impact of demographic, occupational and clinical characteristics on the risk of BI

	Overall sample (*N* = 63,516)	Cumulative incidence of BI (cases = 13,093)	*P* value
Gender			< 0.001
Men	19,344	19.5% (3765)	
Women	44,124	21.1% (9328)	
Age classes			< 0.001
18–29	8188	25.5% (2091)	
30–39	14,187	22.4% (3183)	
40–49	13,239	24.2% (3201)	
50–59	18,944	18.3% (3460)	
≥ 60	8958	12.9% (1158)	
Job title			< 0.001
Physicians	17,209	21.1% (3627)	
Nurse	19,444	24.7% (4803)	
Other health Workers	9333	21.0% (1958)	
Technicians	5530	19.6% (1081)	
Administrative	4462	16.2% (721)	
Previous infection			< 0.001
None	57,404	21.1% (12,143)	
< 1st dose	4610	16.6% (766)	
≥ 1st dose < booster dose	1502	12.3% (184)	
Type of vaccine			< 0.001
BNT162b2	54,710	21.7% (11,855)	
Heterologous	6826	12.0% (822)	
Other	963	20.2% (194)	
Comorbidity			0.848
No	14,191	24.6% (3488)	
Yes	2829	24.7% (700)	
Immunodepression			0.030
No	13,526	24.9% (3367)	
Yes	688	28.6% (197)	

*P* values were computed by the Fisher’s exact test or chi-square test for categorical variables

BI were more frequently recorded in women than men. The cumulative incidence of BI was higher in HW aged < 50 years, decreased in the age class 50–59 years, and nearly halved after 60 years. As regards job title, nurses experienced the highest incidence of BI and administrative staff the lowest. The risk decreased from 21.1% in HW without previous SARS-CoV-2 infection, to 16.6% in HW infected before the 1st dose, and further to 12.3% in those infected after the 1st dose but before the booster one. As regards type of vaccine, the risk of BI was the highest in HW administered BNT162b2 mRNA vaccine, and the lowest in those administered heterologous vaccination. Cumulative incidence of BI was higher in immunosuppressed HW than in the other HW.

**Fig. 1:**
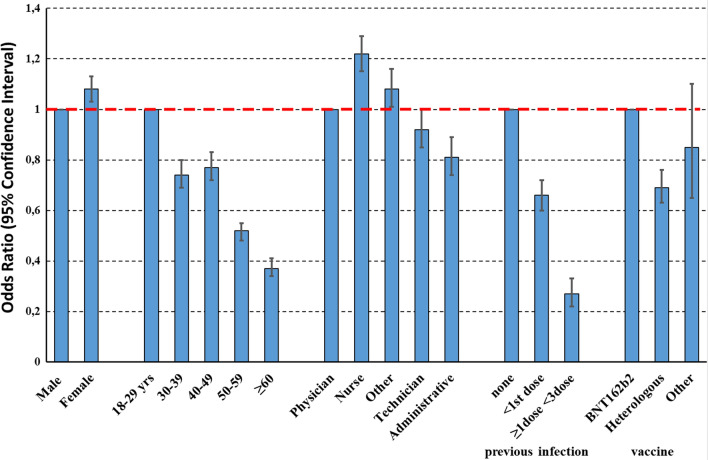
Determinants of BI after the booster dose, investigated by a two‐level logistic regression model, where level‐1 units (HW) were nested into level‐2 units (participating centres)

These results were substantially confirmed in multivariable analysis. In particular, the risk of BI was largely decreased by pre-booster dose infection (OR of infection after 1st dose vs no infection = 0.27, 95% CI 0.22–0.33), older age (OR of > 60 versus 18–29 years = 0.37, 95% CI 0.34–0.41) and type of vaccine (OR of heterologous versus BNT162b2 mRNA vaccination = 0.69, 95% CI 0.63–0.76). Among professions, the risk of BI was the largest in nurses (OR of nurses versus physicians = 1.22, 95% CI 1.15–1.29) and the lowest among administrative staff (OR = 0.81, 95% CI 0.74–0.89) (Fig. 1).

The impact of immunodepression on BI was evaluated on 10,365 HW from 9 centres with available information. When controlling for gender, age, job title, infection before booster dose administration, and type of vaccines, the presence of immunodepression did not significantly affect the risk of BI (OR 1.09, 95% CI 0.88–1.34).

### Determinants of Asymptomatic/Symptomatic Breakthrough Infection

**Fig. 2:**
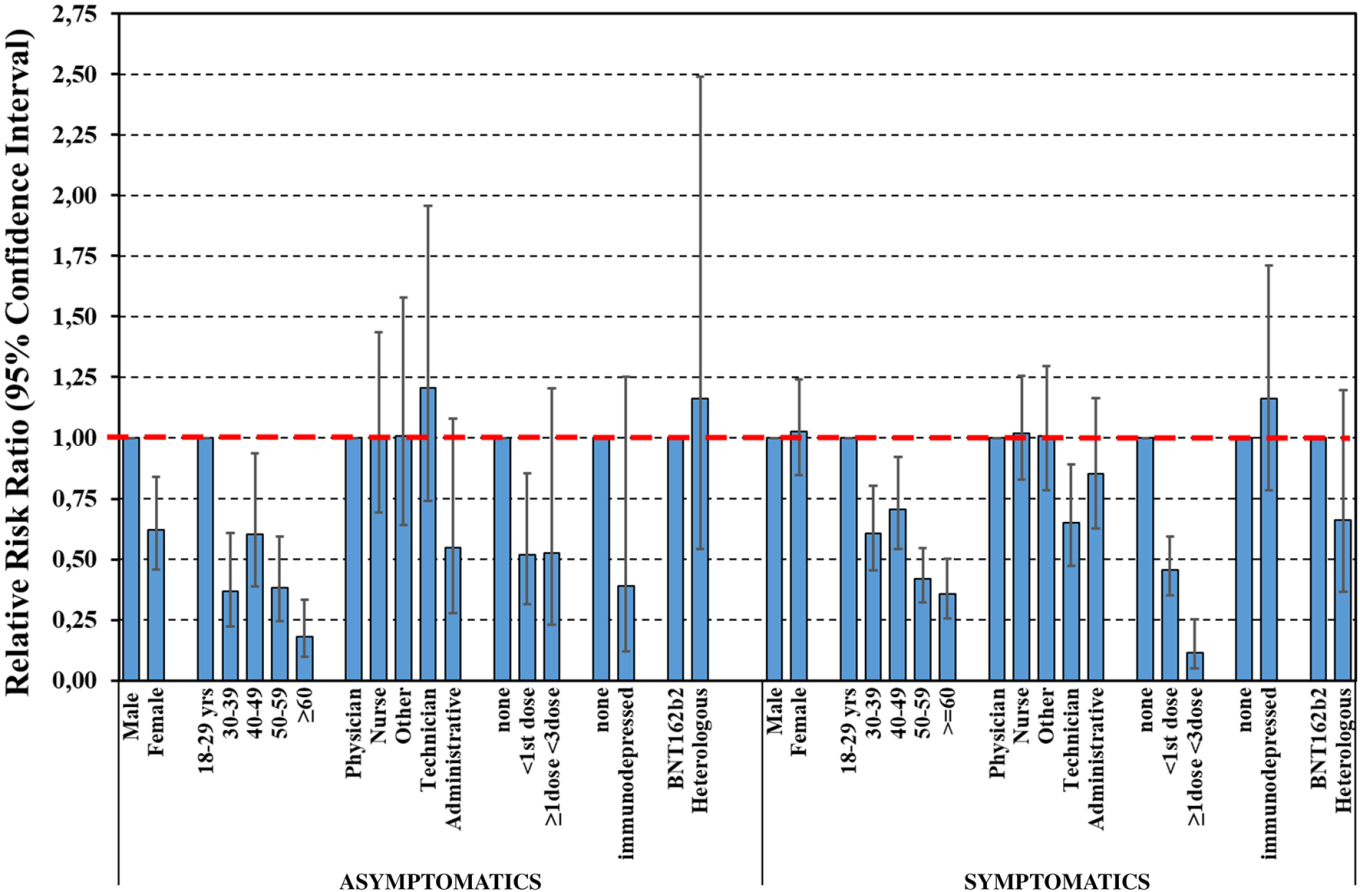
Determinants of BI investigated by a two‐level multinomial logistic regression model (outcome where 0 = no infection, 1 = asymptomatic infection, 2 = symptomatic infection), where level‐1 units (health worker) were nested into level‐2 units (participating centres)

The analysis was carried out on 3100 HW with available information on symptoms during BI from 5 centres (Verona, Padua, Perugia, Northern Barcelona and Slovakia). Overall 2438 HW (78.6%) reported symptomatic infections. Previous infection was negatively associated with asymptomatic infection (RRR of old infection vs no infection = 0.52, 95% CI 0.31–0.85 and RRR of recent infection = 0.53, 95% CI 0.23–1.20) and even more against symptomatic infections (RRR = 0.46, 95% CI 0.35–0.59 and 0.11, 95% CI 0.05–0.25, respectively). With respect to the BNT162b2 mRNA vaccine, heterologous vaccination seemed to provide higher protection against symptomatic infections (RRR = 0.66, 95% CI 0.37–1.20), although the difference was not significant (Fig. 2).

Information concerning hospitalization outcome was available only for 9/14 centres, and 5539 HW. Among them, only 3 (0.05%) were hospitalized following a post-third dose BI.

### Risk of Symptoms During BI as a Function of Time Elapsed from Booster Dose Administration

The risk of symptoms during BI was directly related to the time elapsed since booster dose administration: the proportion of symptomatic infections linearly increased from 70.5% (527/748) to 77.2% (594/769), 80.4% (655/815), 86.2% (662/768) when the time lag increased from 14 to 63 days to 64–93 days, 94–130 days, 131–238 days, respectively (*P* < 0.001). This trend was confirmed in multivariable analysis, controlling for gender, age, job title, infection before booster dose administration, and type of vaccine. With respect to people in the 1st quartile (lag < 64 days), the risk progressively increased from the 2nd quartile (OR 1.55, 95% CI 1.21–1.98) to the 3rd quartile (OR 1.95, 1.52–2.51) and nearly tripled in people in the 4th quartile (117–199 days) (OR 2.76, 2.10–3.64).

## Discussion

The occurrence of BI, even after the second dose of SARS-CoV-2 vaccination, highlighted the urgency of a booster dose, prioritizing high-risk categories, including HW.

Significant variability was observed across centres in the proportion of booster dose administration. These differences could be explained by the different starting periods of the vaccination campaign in various European countries and by different legal obligations for HW [17]. Desye reported that male HW were significantly more likely to accept the COVID-19 vaccine than female HW [18]. In agreement with these results, our study found a slightly higher percentage of vaccinated males than females. At variance with the current literature reporting a higher percentage of booster dose administration among physicians, this survey found a higher percentage of nurses who adhered to vaccination, compared to other patient care professions. Older age and comorbidities are risk factors for COVID-19 severity and poor prognosis [18]. Most HW who consider themselves at risk for COVID-19 *sequelae* are more willing to accept the vaccine. Chew et al. [19] reported that HW willing to vaccinate were more likely to have the perception that the pandemic was severe and that the vaccine was safe and effective. Our results only partly support these findings, as HW with chronic diseases were indeed more likely to have accepted the booster dose, while HW aged over 60 years had the highest percentage of non-vaccinated with booster doses. Unfortunately, we lack data on risk perception and motivations for vaccination, which could have enabled us to further analyse this aspect.

The incidence of BI after the third dose and the trend in antibody titrations are likely the main markers of the effectiveness of SARS-CoV-2 vaccination. In this European multicentric cohort of HW receiving the vaccination against SARS-CoV-2, the overall risk of BI after booster dose was 20.6% (95% CI 20.3–20.9%), more than tenfold higher than BI incidence after full vaccination, as reported in published companion papers also within the framework of the ORCHESTRA Project [4]. To try to explain this striking difference we propose two, possibly synergistic, reasons: the length of the observation period (lag time) and the concomitant pandemic surge during early 2022. The first aspect is associated with the antibody response to vaccination and the titration decay, which progresses as one moves away from the date of vaccination and is intuitively directly proportional to the probability of reinfection. Therefore, short observation periods are likely associated with a lower incidence of BI as was the case of the observation of BI after the second vaccine dose. The second aspect is related to pandemic waves and the spread of variants that may escape the vaccination effect, which is likely more relevant during and after the period when the booster dose was administered. The first studies on BI after booster doses were conducted among Israeli HW due to the early start of all vaccination phases in this country (in July 2021) compared to elsewhere in the world [17]. Among 4,973 HW who received a third BNT162b2 dose since August 2021, Oster et al. found a 0.7% rate of BI, far less the 21.4% reported among HW who received only the two-dose regimen, indicating substantial protection by a third vaccine dose [20]. This finding was confirmed by another Israelian cohort study by Spitzer et al., who recorded five SARS-CoV-2 infections occurring in booster-immunized participants (incidence rate, 12.8 per 100,000 person-days), and 39 occurring in the other HW (incidence rate, 116.1 per 100,000 person-days) [8]. Of note, the study enrolment occurred in August 2021, and the median follow-up was quite short, amounting to 39 days (IQR 35–41 days) for the entire cohort and to 26 days (IQR 21–29 days) for booster-immunized HW. As of December 2021, the Omicron variant spread worldwide, reducing the effectiveness of the then-available vaccinations developed against the wild type. The studies conducted in Israel covered the period August–November, prior to the spread of Omicron [8, 20]; this aspect may be behind the marked difference in the incidence of BI between their studies and our study. The Omicron variant (B.1.1.529 and associated lineages B.1, B1.1, B.2, and B.3) can evade vaccine and natural immunity due in part to several mutations in the spike protein region [21]. Useful insights can be obtained from studies covering BI determinants and regarding vaccination effectiveness.

Therefore, we also investigated the clinical features of BI (symptomatic vs asymptomatic) with regard to several determinants, including previous infection, vaccination approach (homologous vs heterologous) and time elapsed from booster dose administration. In our study, a previous infection protected against asymptomatic infection and even more against symptomatic infections. The risk decreased from 21.1% in HW without previous SARS-CoV-2 infection, to 16.6% in HW infected before the 1st dose, and further to 12.3% in those infected after the 1st dose but before the booster one. These results are consistent with Vivaldi et al. [11] who, in a large prospective population-based study, identified risk factors for SARS-CoV-2 infection after primary and booster vaccinations and found a lower risk of BI in fully vaccinated previously infected subjects. Similar results were found by Lin D. et al. [22] and Andeweg et al. [23]. However, the latter article pointed out that protection against Omicron BA.1 infection, even from a previous infection, was much lower compared to Delta. This comment is coherent with the above remarks about protection throughout the different pandemic waves. According to the above-mentioned studies, Altarawneh et al. reported that hybrid immunity resulting from previous infection and recent booster vaccination conferred the strongest protection [24].

In our research, heterologous vaccination significantly decreased the risk of BI more than homologous BNT162b2 (Pfizer-BioNTech) in the whole sample and also reduced symptomatic infection in a subpopulation analysis, although not significantly. Comparing our results with the available literature, we believe that this aspect deserves further investigation for its implications in guiding current and future vaccination strategies. Indeed, Meggiolaro et al. [7] reported no clear advantage between homologous and heterologous vaccination, particularly on boosting, probably because most of the appraisals have been conducted on mRNA vaccination and data on heterologous vaccination are quite sparse. Au and Cheung conducted a systematic living review and network meta-analysis to evaluate the effectiveness of heterologous and homologous SARS-CoV-2 vaccine regimens with and without boosting in preventing COVID-19-related infection, hospital admission, and death. The authors supported the evidence that an mRNA booster could be recommended to supplement any primary vaccine course. Heterologous and homologous three-dose regimens work comparably well in preventing COVID-19 infections, even against different variants.

The effectiveness of three-dose vaccine regimens against COVID-19-related death remains uncertain [25, 26]. The time elapsed since booster dose administration can also affect the BI severity, as in the present study the risk of symptoms during BI linearly and significantly increased with time elapsed since booster dose administration both in univariable and multivariable analyses. In their systematic review and meta-analysis, Meggiolaro et al. confirmed that a primary vaccination course does not adequately protect against Omicron because the probabilities of symptomatic infection and related hospitalization are nearly 50% for vaccinated compared to unvaccinated. On the other hand, one additional booster dose decreases by 69% the risk of symptomatic Omicron infection and by 88% the risk of hospitalization as compared to unvaccinated at a maximum follow-up of 5 months. Moreover, the subgroup analysis does not suggest waning of booster dose effectiveness after five months. However, the evidence on long-term effectiveness is still limited. In our research, we consider only the presence vs. absence of symptoms. Overall, for all centres with available data, symptomatic BI were greater. Nearly 80% of SARS-CoV-2 infections in this cohort were symptomatic, similar to the proportion observed in other studies of vaccine BI [8]. This finding can certainly be related to the prevalence of the Omicron variant, and its high contagiousness and widespread.

The 9-month observation time covered a period in which a series of SARS-CoV-2 variants were predominant in Europe, in particular the Omicron ones, and the results of this study suggest that boosters continued to provide protection against severe illness despite viral evolution. On the other hand, no deaths of post-third dose BI were reported in the present study, and even hospitalization was extremely rare (< 0.1%). Regarding this outcome, Andrews et al. reported that the absolute effectiveness of a BNT162b2 booster against hospitalization or death ranged from around 97 to 99% in all age groups irrespective of the primary course, providing real-world evidence of substantially increased protection from the booster vaccine dose against severe disease [27]. According to the meta-analysis by Flacco et al. [28], vaccinated subjects showed a significantly lower likelihood of reinfection, as compared to the unvaccinated. Notably, the results did not change up to 12 months of follow-up irrespective of the number of vaccine doses, in studies that adjusted for potential confounders, adopting different reinfection definitions, and with different predominant strains. Once re-infected, vaccinated subjects were also significantly less likely to develop a severe disease (OR 0.45; 95% CI 0.38–0.54). The authors reported that their meta-analysis provides solid evidence of a more robust protection of hybrid vs. natural immunity, which may persist during Omicron waves and up to 12 months. Furthermore, the second main finding by Flacco et al. was the significant reduction of the risk of hospitalization due to severe COVID-19 that was observed among the vaccinated subjects, either receiving one or more doses. Nevertheless, the authors also underlined that most of the studies included in the meta-analyses were carried out before the emergence of Omicron strain. Therefore, their finding requires confirmation stemming from more recent data with longer follow-up, as the significant increase in the number of reinfections during the Omicron wave and the consequences on the healthcare systems still need to be carefully evaluated.

Further observations may concern other characteristics investigated in this research, such as age, job title and comorbidities. In line with the former survey and available literature [4, 11], we found that older age was at lower risk of post-booster dose BI incidence. As hypothesized in our previous study, this negative correlation could be possibly due to increased social contacts as well as to the assignment of younger HW to higher risk wards [10]. Job title is another frequently investigated determinant in studies of SARS-CoV-2 infections, especially those on HW. In these regards, the results are often conflicting, and their interpretation is not always smooth [4, 29]. In this survey, nurses resulted in the higher risk category for BI. As regards the role of comorbidities, we found an association between BI post-booster dose and pre-existing immunodepression.

Future researches should focus on BI after 4th dose and its determinants, along with their trend and relations with time variables across the epidemics.

### Strengths and Limitations

Major strengths of the paper are the sample size, the largest—to the best of our knowledge—in the available published literature on HW, and the multicentric recruitment. The size of the study, the representativeness of the case list, and our inclusion of three of the most widely used vaccines for primary and booster vaccinations together increase the generalisability of our findings. This is coupled with a solid methodology in conducting the research, also supported by laboratory data from certified laboratory centres. These aspects lend power to the study and provide good stability of results and generalizability.

In addition, the time frame of observation was wide and enabled evaluating the entire time window before the second booster dose. It is also an ongoing study that will be followed by an appraisal of BI after the fourth dose.

The study also has some limitations to be disclosed. Data lacking on risk perception and motivations for vaccination can be considered a limitation. The analysis of clinical data was only conducted on 15% of the population (13,000 HW), albeit a large one, for which individual data were available. As regards comorbidity outcome, only data on the presence or absence were available and, in any case, just for some centres. This hindered the possibility to evaluate the weight of this determinant on the incidence of BI. Nevertheless, immunodepression emerged as a risk factor for BI after booster dose.

The multicentricity also presented some critical issues, such as those related to the different screening approaches in the several centres in terms of the sampling time, which could lead to an overall underestimation of the incidence of BI. For the same reason, data on post-dose booster antibody titrations were also not available.

## Conclusion

The risk of SARS-CoV-2 BI after the booster dose is significantly reduced by previous infection, heterologous vaccination and older age. Nurses are at higher risk of infection among HW, in a context of different findings from the literature. Immunodepression is associated with increased BI incidence after the booster dose. The time elapsed from booster dose administration affects BI severity, confirming the usefulness of booster doses and its relevance for public health. Further researches should focus on BI after 4^th^ dose and its determinants, along with their trend and relations with time variables across the epidemics.

## Supplementary Information

Below is the link to the electronic supplementary material.

Supplementary file1 (DOCX 34 KB)

## Data Availability

The datasets generated during the current study are not publicly available because they contain sensitive data to be treated under data protection laws and regulations. Appropriate forms of data sharing can be arranged after a reasonable request to the first author.

## Abbreviations

AHR: Adjusted hazard ratio
BI: Breakthrough infections
BMI: Body mass index
HW: Health workers
IQR: Interquartile range
OR: Odds ratio
RRR: Relative risk ratio
