# Supplementary material for: Incidence and Determinants of Symptomatic and Asymptomatic SARS-CoV-2 Breakthrough Infections After Booster Dose in a Large European Multicentric Cohort of Health Workers-ORCHESTRA Project
Source: J Epidemiol Glob Health. 2023 Jul 22;13(3):577–88. doi: 10.1007/s44197-023-00139-8 (PMC10468456; doi:10.1007/s44197-023-00139-8)
Supplement: Supplementary file 1 — Supplementary file1 (DOCX 34 KB) [file 44197_2023_139_MOESM1_ESM.docx]

**Supplementary**

**Table 1S.** Breakthrough infections, demographic, and occupational characteristics of Health Workers from 14 European centers. Data are reported as counts with percentages in parenthesis.

| **Center** | **HW Full sample** | **HW receiving booster dose** | **BI after booster dose** | **Sex** | | **Job Title** | | | | | **Age Classes** | | | | |
| --- | --- | --- | --- | --- | --- | --- | --- | --- | --- | --- | --- | --- | --- | --- | --- |
|  |  |  |  | **Men** | **Women** | **Physician** | **Nurse** | **Other** | **Technician** | **Administrative** | **<30** | **30–39** | **40–49** | **50-59** | **≥60** |
| **Turin** | 10,748 | 9,816  (91.3) | 1,712  (17.4) | 2,799 (28.6) | 6,975 (71.4) | 1,862  (21.6) | 3,142  (36.5) | 1,158  (13.4) | 1,501  (17.4) | 957  (11.1) | 1,651 (16.8) | 1,529 (15.6) | 1,941 (19.8) | 3,226 (32.9) | 1,469  (15.0) |
| **Brescia** | 8,903 | 8,212  (92.2) | 1,691  (20.6) | 2,255 (27.5) | 5,957 (72.5) | 2,432  (29.6) | 2,669  (32.5) | 1,537  (18.7) | 664  (8.1) | 910  (11.1) | 871 (10.6) | 1,964 (23.9) | 1,703 (20.7) | 2,631 (32.0) | 1,043  (12.7) |
| **Verona** | 6,377 | 5,535  (86.8) | 1,721  (31.1) | 1,641 (29.6) | 3,894 (70.4) | 1,764  (31.9) | 1,994  (36.0) | 840  (15.2) | 514  (9.3) | 423  (7.6) | 950 (17.2) | 1,237  (22.3) | 1,183 (21.4) | 1,703 (30.8) | 462  (8.3) |
| **Padua** | 8,511 | 6,548  (76.9) | 1,943  (29.7) | 1,976 (30.2) | 4,572 (69.8) | 2,065  (31.6) | 2,451  (37.4) | 948  (14.5) | 591  (9.0) | 491  (7.5) | 891 (13.6) | 1,512 (23.1) | 1,203 (18.4) | 2,186 (33.4) | 7576  (11.5) |
| **Trieste** | 7,959 | 6,096  (76.6) | 1,761  (29.7) | 1,933 (31.7) | 4,163 (68.3) | 1,326  (21.7) | 2,190  (35.9) | 1,248  (20.5) | 882  (14.5) | 450  (7.4) | 585  (9.6) | 1,169 (19.2) | 1,346 (22.1) | 2,018 (33.1) | 978  (16.0) |
| **Modena** | 5,267 | 5,003  (94.9) | 695  (13.9) | 1,463 (29.2) | 3,540 (70.8) | 1,417  (31.1) | 1,765  (38.7) | 951  (20.9) | 160  (3.5) | 266  (5.8) | 705 (14.1) | 1,385 (27.7) | 1,079 (21.6) | 1,267 (25.3) | 567 (11.3) |
| **Bologna** | 7,597 | 7,054  (92.8) | 1,358  (19.2) | 2,242 (31.8) | 4,812 (68.2) | 2,341  (33.3) | 2,421  (34.4) | 1,293  (18.4) | 641  (9.1) | 344  (4.9) | 889 (12.6) | 2,067 (29.3) | 1,475 (20.9) | 1,789 (25.4) | 834  (11.8) |
| **Perugia** | 3,805 | 2,191  (57.6) | 317  (14.5) | 760  (34.7) | 1,431 (65.3) | 612  (28.4) | 834  (38.6) | 304  (14.1) | 275  (12.7) | 133  (6.2) | 37  (1.7) | 364 (16.6) | 435 (19.9) | 725 (33.1) | 630  (28.7) |
| **Bari** | 6,196 | 5,641  (91.0) | 862  (15.3) | 2,218 (39.3) | 3,423 (60.7) | 2,728  (48.4) | 1,582  (28.0) | 794  (14.1) | 198  (3.5) | 339  (6.0) | 720 (12.8) | 1,464 (25.9) | 953 (16.9) | 1,478 (26.2) | 1,026  (18.2) |
| **Oviedo** | 8,226 | 2,559  (31.1) | 599  (23.4) | 548  (21.4) | 2,010  (78.6) | --- | --- | --- | --- | 7  (100.0) | 82  (3.2) | 430  (16.8) | 818 (32.0) | 739 (28.9) | 490 (19.1) |
| **Barcelona** | 848 | 513  (60.5) | 71  (13.8) | 125  (24.4) | 388  (75.6) | 166  (34.9) | 162  (34.0) | 78  (16.4) | 0  (0.00) | 70  (14.7) | 54  (10.5) | 74  (14.4) | 168 (32.8) | 122 (23.8) | 95  (18.5) |
| **Munich** | 3,282 | 3,259  (99.3) | 214  (6.6) | 856  (26.3) | 2,398  (73.7) | --- | ---- | --- | --- | ---- | 669 (20.5) | 876 (26.9) | 640 (19.6) | 700 (21.5) | 374 (11.5) |
| **Slovakia** | 1,072 | 500  (46.6) | 145  (29.0) | 414  (82.8) | 86  (17.2) | 75  (15.0) | 164  (32.9) | 171  (34.3) | 37  (7.4) | 52  (10.4) | 56  (11.2) | 60  (12.0) | 139 (27.8) | 144 (28.8) | 101  (20.2) |
| **Romania** | 1,672 | 589  (35.2) | 4  (0.7) | 114  (19.4) | 475  (80.6) | 421  (71.5) | 70  (11.9) | 11  (1.9) | 67  (11.4) | 20  (3.4) | 28  (4.7) | 56  (9.5) | 156 (26.5) | 216 (36.7) | 133  (22.6) |
| **Total** | **80,463** | **63,516**  **(78.9)** | **13,093**  **(20.6)** | **19,344**  **(30.5)** | **44,124**  **(69.5)** | **17,209 (30.7)** | **19,444**  **(34.7)** | **9,333**  **(16.7)** | **5,530**  **(9.9)** | **4,462**  **(8.0)** | **8,188 (12.9)** | **14,187 (22.3)** | **13,239 (20.8)** | **18,944 (29.8)** | **8,958 (14.1)** |

**Table 2S.** Distribution of Type of vaccine by center

| **Center** | **BNT162b2 (Pfizer– BioNTech)** | **Heterologous** | **mRNA-1273 (Moderna)** | **ChAdOx1 nCoV-19 (Oxford–AstraZeneca)** | **Ad26.COV2.S (Johnson)** | **Other** |
| --- | --- | --- | --- | --- | --- | --- |
| **Turin** | 8,447 (86.07%) | 1,367 (13.93%) | --- | --- | --- | --- |
| **Brescia** | 6,501 (80.83%) | 1,513 (18.81%) | 29 (0.36%) | --- | --- | --- |
| **Verona** | 5,185 (100.00%) | --- | --- | --- | --- | --- |
| **Padua** | 6,490 (99.11%) | 55 (0.84%) | 3 (0.05%) | --- | --- | --- |
| **Trieste** | 5,550 (96.17%) | --- | 202 (3.50%) | 16 (0.28%) | 3 (0.05%) | --- |
| **Modena** | 2,927 (58.52%) | 2,034 (40.66%) | 41 (0.82%) | --- | --- | --- |
| **Bologna** | 6,707 (95.94%) | 284 (4.06%) | --- | --- | --- | --- |
| **Perugia** | 2,191 (100.00%) | --- | --- | --- | --- | --- |
| **Bari** | 5,641 (100.00%) | --- | --- | --- | --- | --- |
| **Oviedo** | 1,801 (72.74%) | 76 (3.07%) | 599 (24.19%) | --- | --- | --- |
| **Northern Barcelona** | 123 (24.02%) | 363 (70.90%) | 26 (5.08%) | --- | --- | --- |
| **Munich** | 2,122 (65.23%) | 1,099 (33.78%) | 31 (0.95%) | --- | --- | 1 (0.03%) |
| **Slovakia** | 469 (96.70%) | 10 (2.06%) | 6 (1.24%) | --- | --- | --- |
| **Romania** | 556 (94.72%) | 25 (4.26%) | 6 (1.02%) | --- | --- | --- |
| **Total** | **54,710 (87.54%)** | **6,826 (10.92%)** | **943 (1.51%)** | **16 (0.03%)** | **3 (0.00)** | **1 (0.00)** |
